# Supplementary material for: Wavelet Analysis of Dual‐fMRI‐Hyperscanning Reveals Cooperation and Communication Dependent Effects on Interbrain Neuronal Coherence
Source: Hum Brain Mapp. 2025 Sep 24;46(14):e70355. doi: 10.1002/hbm.70355 (PMC12460188; doi:10.1002/hbm.70355)
Supplement: Supplementary file 1 — Figure S1: Bar graphs visualizing mean accuracy during both synchronization tasks (Cooperation and Communication), plotted per session. Error bars depict standard error. Asterisks indicate significance at p < 0.05 (*) and p < 0.001 (**). Table S2: Regions of interest showing significant coherence differences (F test). ROIs are labeled following the Harvard–Oxford cortical structural atlas. #ROI specifies the ROI number based on the Kong‐17 networks order. Table S3: Regions of interest showing significant contrast‐specific coherence differences compared to cooperation (post hoc paired t test). ROIs are labeled following the Harvard‐Oxford cortical structural atlas. #ROI specifies the ROI number based on the Kong‐17 networks order. [file HBM-46-e70355-s001.docx]

**Supporting Information**

**Wavelet Analysis of Dual-fMRI-Hyperscanning Reveals Cooperation and Communication Dependent Effects on Interbrain Neuronal Coherence.**

Rik Sijben^1^, Robert Friedmann^1^, Lucia Hernandez-Pena^2,3^, Rea Rodriguez-Raecke^1^

^1^Brain Imaging Facility, Interdisciplinary Center for Clinical Research (IZKF), RWTH Aachen University, Aachen, Germany.

^2^Department of Psychiatry, Psychotherapy and Psychosomatics, Faculty of Medicine, RWTH Aachen University, Aachen, Germany.

^3^JARA – Translational Brain Medicine, Aachen, Germany

Corresponding author: Dr. rer. medic. Rik Sijben

**Email:**  [rsijben@izkf.rwth-aachen.de](mailto:rsijben@izkf.rwth-aachen.de)

**Content:**

Figure S1

Tables S2 to S3


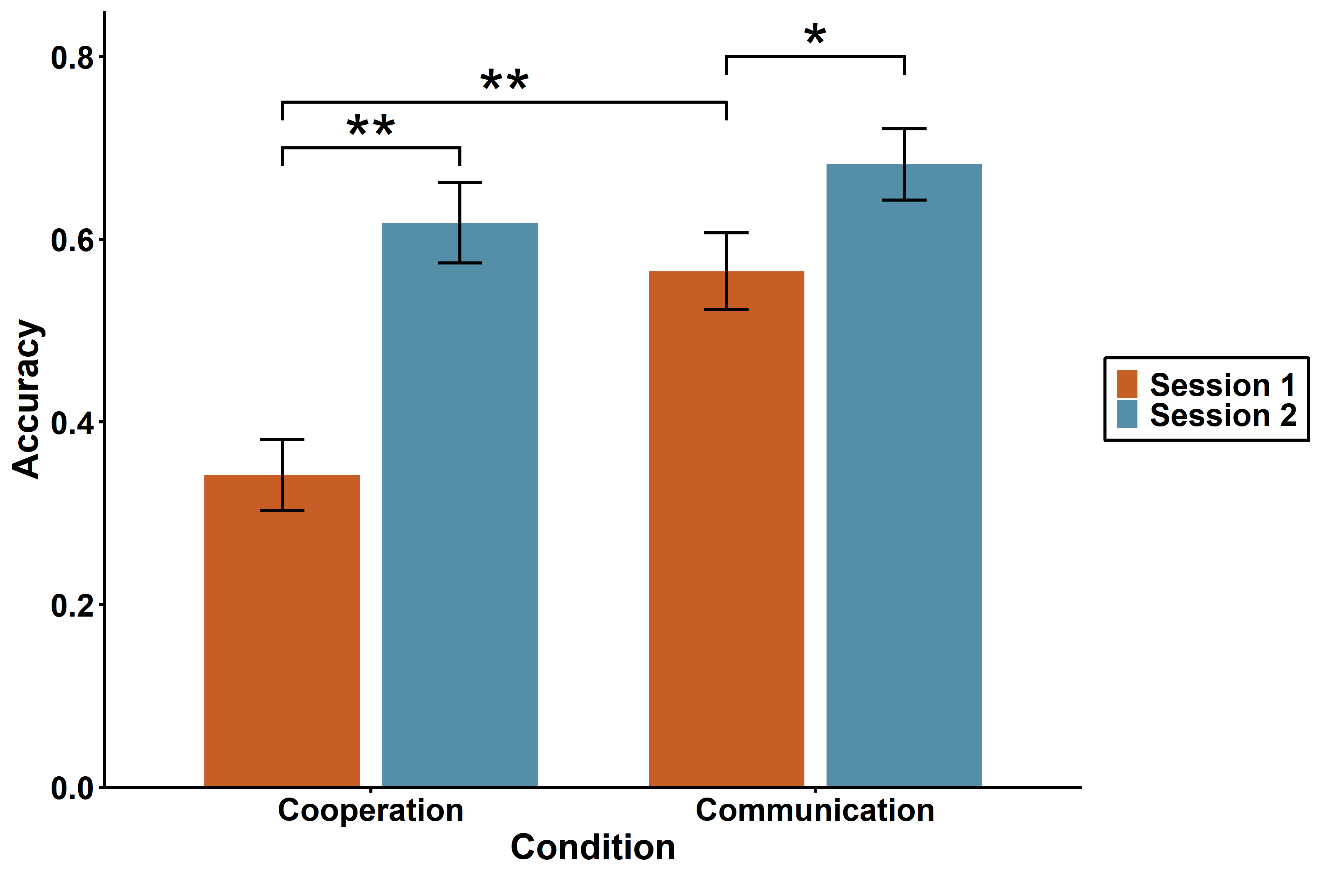


Figure S1. Bar graphs visualizing mean accuracy during both synchronization tasks (Cooperation and Communication), plotted per session. Error bars depict standard error. Asterisks indicate significance at p < .05 (*) and p < .001 (**).

Table S2. Regions of interest showing significant coherence differences (F-test). ROIs are labeled following the Harvard-Oxford cortical structural atlas. #ROI specifies the ROI number based on the Kong-17 networks order.

| [#ROI] ROI Label | Centroid Coordinates | | | F | *df* | P-value (FDR) |
| --- | --- | --- | --- | --- | --- | --- |
|  | X | Y | Z |  |  |  |
| [#149] L Precentral Gyrus | -36 | -20 | 64 | 11.20 | 3, 72 | < .001 |
| [#165] L Precentral Gyrus | -40 | -14 | 48 | 6.22 | 3, 84 | .019 |
| [#183] L Precentral Gyrus | -40 | -2 | 52 | 5.24 | 3, 66 | .040 |
| [#152] L Postcentral Gyrus | -48 | -18 | 54 | 6.73 | 3, 78 | .041 |
| [#151] L Postcentral Gyrus | -38 | -24 | 52 | 5.40 | 3, 84 | .033 |
| [#285] R Supp. Motor Cortex | 6 | -2 | 66 | 6.56 | 3, 78 | .015 |
| [#086] L Ant. Cingulate Gyrus | -6 | 0 | 40 | 8.45 | 3, 66 | .003 |
| [#085] L Paracingulate Gyrus | -6 | 10 | 48 | 7.71 | 3, 78 | .004 |
| [#287] R Ant. Cingulate Gyrus | 8 | 2 | 42 | 5.13 | 3, 78 | .040 |
| [#295] R Ant./ Paracingulate Gyrus | 8 | 18 | 36 | 5.35 | 3, 84 | .033 |
| [#066] L Ang. Gyrus/Lat. Occip. Cortex | -48 | -60 | 46 | 5.86 | 3, 66 | .025 |
| [#142] L Heschl's Gyrus | -50 | -10 | 0 | 13.99 | 3, 69 | < .001 |
| [#137] L Heschl's Gyrus | -36 | -24 | 10 | 6.03 | 3, 81 | .040 |
| [#141] L Planum Temporale | -56 | -22 | 8 | 29.62 | 3, 84 | < .001 |
| [#140] L Planum Temporale | -58 | -36 | 16 | 6.09 | 3, 72 | .021 |
| [#100] L Front. Operculum Cortex | -32 | 18 | 8 | 8.80 | 3, 72 | .002 |
| [#027] L Post. Sup. Temporal Gyrus | -60 | -34 | 4 | 16.31 | 3, 87 | < .001 |
| [#145] L Ant. Sup. Temporal Gyrus | -60 | -12 | -2 | 25.05 | 3, 72 | < .001 |
| [#048] L Mid. Temporal Gyrus | -52 | -44 | 4 | 4.91 | 3, 75 | .048 |
| [#050] L Temporal Pole | -52 | 6 | -12 | 10.21 | 3, 63 | .003 |
| [#340] R Heschl's gyrus | 54 | -14 | 6 | 36.61 | 3, 78 | < .001 |
| [#339] R Planum Temporale | 60 | -24 | 10 | 36.11 | 3, 78 | < .001 |
| [#343] R Post. Sup. Temporal Gyrus | 62 | -18 | 0 | 31.69 | 3, 87 | < .001 |
| [#241] R Post. Sup. Temporal Gyrus | 50 | -34 | 2 | 9.59 | 3, 84 | < .001 |
| [#342] R Post. Sup. Temporal Gyrus | 64 | -34 | 10 | 6.12 | 3, 87 | .019 |
| [#210] R Ant. Sup. Temporal Gyrus | 54 | -4 | -14 | 6.17 | 3, 84 | .019 |
| [#223] R Post. Mid. Temporal Gyrus | 64 | -24 | -8 | 5.14 | 3, 75 | .040 |
| [#211] R Post. Temporal Fusiform Cortex | 40 | -14 | -32 | 5.81 | 3, 84 | .023 |
| [#180] L Occipital Fusiform Gyrus | -24 | -72 | -10 | 7.65 | 3, 87 | .023 |
| [#384] R Occipital Fusiform Gyrus | 24 | -74 | -10 | 6.72 | 3, 87 | .028 |

**Table S3.** Regions of interest showing significant contrast-specific coherence differences compared to cooperation (post-hoc paired T-test). ROIs are labeled following the Harvard-Oxford cortical structural atlas. #ROI specifies the ROI number based on the Kong-17 networks order.

| **Contrast**  [#ROI] ROI Label | Centroid Coordinates | | | T | *df* | P-value (FDR) |
| --- | --- | --- | --- | --- | --- | --- |
|  | X | Y | Z |  |  |  |
| **Cooperation > Solo** |  |  |  |  |  |  |
| [#149] L Precentral Gyrus | -36 | -20 | 64 | 4.62 | 25 | < .001 |
| [#165] L Precentral Gyrus | -40 | -14 | 48 | 2.96 | 29 | .018 |
| [#183] L Precentral Gyrus | -40 | -2 | 52 | 3.19 | 28 | .010 |
| [#151] L Postcentral Gyrus | -38 | -24 | 52 | 2.61 | 28 | .043 |
| [#085] L Paracingulate Gyrus | -6 | 10 | 48 | 3.64 | 27 | .003 |
| [#066] L Ang. Gyrus/Lat. Occip. Cortex | -48 | -60 | 46 | 3.00 | 25 | .009 |
| [#285] R Supp. Motor Cortex | 6 | -2 | 66 | 3.50 | 26 | .005 |
| [#100] L Front. Operculum Cortex | -32 | 18 | 8 | 2.87 | 28 | .023 |
| [#180] L Occipital Fusiform Gyrus | -24 | -72 | -10 | 2.99 | 29 | .017 |
| [#384] R Occipital Fusiform Gyrus | 24 | -74 | -10 | 2.69 | 29 | .036 |
|  |  |  |  |  |  |  |
| **Cooperation > Competition** |  |  |  |  |  |  |
| [#066] L Ang. Gyrus/Lat. Occip. Cortex | -48 | -60 | 46 | 3.86 | 25 | .002 |
|  |  |  |  |  |  |  |
| **Competition > Cooperation** |  |  |  |  |  |  |
| [#086] L Ant. Cingulate Gyrus | -6 | 0 | 40 | -2.99 | 25 | .019 |
| [#211] R Post. Temporal Fusiform Cortex | 40 | -14 | -32 | -3.71 | 29 | .003 |
| [#050] L Temporal Pole | -52 | 6 | -12 | -3.29 | 23 | .005 |
| **Cooperation > Communication** |  |  |  |  |  |  |
| [#180] L Occipital Fusiform Gyrus | -24 | -72 | -10 | 2.66 | 29 | .019 |
| [#384] R Occipital Fusiform Gyrus | 24 | -74 | -10 | 2.33 | 29 | .040 |
|  |  |  |  |  |  |  |
| **Communication > Cooperation** |  |  |  |  |  |  |
| [#141] L Planum Temporale | -56 | -22 | 8 | -6.90 | 29 | < .001 |
| [#140] L Planum Temporale | -58 | -36 | 16 | -3.18 | 28 | .011 |
| [#050] L Temporal Pole | -52 | 6 | -12 | -5.30 | 27 | < .001 |
| [#142] L Heschl's Gyrus | -50 | -10 | 0 | -5.10 | 27 | < .001 |
| [#137] L Heschl's Gyrus | -36 | -24 | 10 | -3.67 | 27 | .003 |
| [#145] L Ant. Sup. Temporal Gyrus | -60 | -12 | -2 | -5.44 | 27 | < .001 |
| [#027] L Post. Sup. Temporal Gyrus | -60 | -34 | 4 | -5.43 | 29 | < .001 |
| [#048] L Mid. Temporal Gyrus | -52 | -44 | 4 | -3.88 | 25 | .002 |
| [#339] R Planum Temporale | 60 | -24 | 10 | -6.87 | 27 | < .001 |
| [#340] R Heschl's gyrus | 54 | -14 | 6 | -6.86 | 29 | < .001 |
| [#210] R Ant. Sup. Temporal Gyrus | 54 | -4 | -14 | -2.90 | 29 | .021 |
| [#343] R Post. Sup. Temporal Gyrus | 62 | -18 | 0 | -6.04 | 29 | < .001 |
| [#241] R Post. Sup. Temporal Gyrus | 50 | -34 | 2 | -3.97 | 29 | .001 |
| [#342] R Post. Sup. Temporal Gyrus | 64 | -34 | 10 | -3.74 | 29 | .002 |
| [#223] R Post. Mid. Temporal Gyrus | 64 | -24 | -8 | -2.73 | 26 | .034 |
